# What’s the name? Weight stigma and the battle against obesity

**DOI:** 10.1186/s13052-020-00821-8

**Published:** 2020-05-13

**Authors:** Rita Tanas, Sergio Bernasconi, Maria Marsella, Giovanni Corsello

**Affiliations:** 1 Advisor of the Adolescence Study Group, Italian Pediatric Society, Ferrara, Italy; 2 Microbiome Researc Hub Università, Parma, Italy; 3 UOC di Pediatria, Azienda Ospedaliera San G. Moscati, Avellino, Italy; 4 Dipartimento di Scienze per la Promozione della Salute e Materno Infantile “G. D’Alessandro” Università, Palermo, Italy

**Keywords:** Obesity, Weight stigma, Language, Terminology, Weight management

## Abstract

Childhood obesity has spread worldwide, it is on the rise, starts earlier and is more severe, despite all treatment attempts.

According to recent studies, weight stigma is a factor that can hinder the success of therapies. Healthcare workers, mainly paediatricians, need to feel the urgency of anti-stigma training. The use of non-stigmatizing terminologies and images in various areas (school, sports clubs, healthcare, media, society in general) can improve disease management.

## Background

In recent years several epidemiological studies have reported an increase in childhood obesity worldwide. This epidemic is known to be related to complications, which are already seen in children and adolescents, and represents a significant risk factor for adult obesity, which increases morbidity and mortality.

In other words, scientific evidence demonstrates that becoming obese in childhood has significant consequences on physical and mental health and on quality of life. Furthermore, obesity increases the incidence and worsens progression of both chronic and acute diseases.

These considerations have stimulated numerous projects for more effective therapeutic interventions, and, moreover, for prevention of the disease. However, the results obtained so far are not satisfactory [1]. We must, therefore, ask ourselves where are we wrong and what can we do more and better [2]. It is not easy to answer these questions, because obesity is a multifactorial disease, that we still are not able to define completely, and it is probable that we use the term obesity to indicate a variety of subcategories, which would require specific and personalized interventions.

One of the factors that can influence the course of the disease has only recently been considered by researchers: STIGMA.

Obesity stigma consists in attributing a priori negative meanings to the overweight condition. Whether it manifests itself openly, *explicit*, or it remains unconscious, *implicit*, stigma leads to considering overweight people “lazy, sloppy, unintelligent and unattractive”. Stigmatization occurs everywhere: in the family, at school and in the healthcare setting [3].

In the family it begins very early in life, already in pre-school age, and leads parents to take on restrictive and dangerous food habits, even discriminating their offspring. Social behavioural studies report that obese children at age 6 are ignored by their peers, while severely obese children are rejected.

Healthcare stigma is described in all areas, from primary to speciality care, even in obesity care researchers.

It is often unconscious and increases with progression of professional age. Its strongest determinant is the attribution of obesity mostly to personal responsibility, despite the numerous and complex causes of weight gain.

It is common belief that weight depends mostly upon individual responsibility and control of diet and physical activity, and that a high body mass index indicates bad habits, which lead to poor health, for which the obese individual is ultimately responsible for. This prejudice perpetuates and worsens stigma and obesity itself.

Weight stigma has become stronger and more frequent than race and sexual orientation stigma and continues to increase.

Over time it is internalized, especially in childhood, so that the victims themselves become creators of self-derision, which grows to spectacularization, increasing dissatisfaction towards their bodies, depression, eating disorders, bullying and even mortality [4].

Potential harmful effects of stigma are not only psychological (depression, anxiety, low self-esteem, suicidal ideation, dissatisfaction of the body), but also physical (unhealthy eating behaviours: skipping meals, very low calorie diets < 800 cal / day, ready-made low-calorie meals; or extreme eating behaviours: fasting, vomiting, use of drugs and refusal of physical activity).

Stigma is however still considered useful, even by healthcare professionals to motivate patients towards the adoption of weight loss favouring behaviours. Unfortunately it brings to faster and less convincing visits, therefore less effective messages, and greater physical distance between physician and patient. It reduces or delays the request for treatment and the intention to change behaviours, reducing self-efficacy.

It would be useful for healthcare professionals and paediatricians to perceive the urgency of creating school, university and post-university training projects to spread knowledge about stigma, trying to reduce it in all fields. We must start from our clinical environment to improve the quality of care and maybe obtain better results on care acceptance, care adherence and health improvement [5, 6].

Mocking a person who has been derided since the early years of his life is very easy, and is often involuntary, but it can have devastating consequences. Where should we begin?

One of the ways to go is to **change the images** that the media, but sometimes we too, associate with obesity, and involve journalists, directors, colleagues, students, nurses in the use of more positive or at least neutral images.

The other way is to **change words** in communication. Dedicated literature invites us to use terms that do not offend families and children/teens and motivate them to seek treatment; for example, no longer use and allow others to use the term obese children, but children with obesity in complete respect of their personality [7]!

Keeping these aspects in mind, one of us (RT) proposed to paediatricians, who joined the obesity study group of the Society of Paediatric Endocrinology and Diabetology, to administer a short questionnaire to parents of children with overweight/obesity and possibly also to their children aged over 11 years.

Its first objective is to understand whether there are terms that are simultaneously motivating and non-offensive to be adopted in paediatric clinical practice.

The second objective is to widen, as much as possible, the awareness of professionals, their collaborators and young trainees on the professional weight stigma!

There are numerous similar studies in literature from 2003 with questionnaires offered to adults with and without obesity, adolescents, parents of children with obesity, healthcare workers: physicians and dieticians, have tested words in English and, more recently, also in other languages. However, we were not able to find any in Italian [8, 9].

Acceptance and adhesion to the study of professionals was very good in all regions of Italy: 12 second level centres for the care of childhood obesity and a group of 10 family paediatricians, but we still continue to receive requests to participate. From an initial analysis of the first 150 questionnaires administered to parents and children with an average age of 13 years, some preliminary data emerged, which can be summarized as follows:

1. The most offensive words are: obesity and fat.
2. There is no single term shared by families and children that can replace the offensive ones.

Several terms were proposed as most motivating, such as *weight higher than normal, inadequate weight, very robust, unhealthy weight*. Therefore the practical advice that can be given to healthcare professionals in contact with families and patients is to use the terminology preferred by the family.

In the final diagnostic report the value of the BMI percentile or z-score might be adopted, omitting the term obesity and specifying at the bottom of the page the cut-offs for its evaluation.

## Conclusions

The use of non-stigmatizing terminology on behalf of healthcare professionals appears quite gratifying to patients and families. This could help in creating a better empathetic environment, which is undoubtedly useful in the complex and difficult management of a multiform disease, like obesity [10]. This attitude should be adopted at a more general level (school, sports clubs, society in general), so that overweight children/teens feel more accepted and supported in the management of their disease, as it happens with many other chronic situations. Finally, it could trigger reduction of universal stigma of obesity, thus improving the quality of life of these patients.

## Data Availability

There are not data and materials.
